# A First Attempt into the Production of Acylglycerol Mixtures from Echium Oil

**DOI:** 10.3389/fbioe.2015.00208

**Published:** 2016-01-19

**Authors:** Luis Vázquez, Alejandro Jordán, Guillermo Reglero, Carlos F. Torres

**Affiliations:** ^1^Departamento de Producción y Caracterización de Nuevos Alimentos, Instituto de Investigación en Ciencias de la Alimentación (CSIC–UAM), Universidad Autónoma de Madrid, Madrid, Spain; ^2^IMDEA-Food Institute, CEI UAM-CSIC, Madrid, Spain

**Keywords:** echium oil, glycerolysis, lipase, self-emulsifying, monoacylglycerol

## Abstract

Enzymatic glycerolysis of Echium oil (*Echium plantagineum*) has been carried out in the presence of four commercial lipases. Different pretreatments of the reaction mixture, such as high pressure homogenization and addition of food grade monoolein as an emulsifier, were evaluated to test their influence on the glycerolysis reaction. In addition, the impact of reducing temperature and the utilization of a solvent generally recognized as safe as a flavoring agent, such as limonene, were also investigated. Conversion of ca. 60–70% of triacylglycerols and production of ca. 25–30% of monoacylglycerols (MAGs) were attained. Finally, at the best reaction conditions, the glycerolysis reaction was scaled up at pilot plant and the product mixture obtained was fractionated via molecular distillation. From this stage, two products were attained: a distillate containing 80% of MAGs and a residue containing approximately 50% of diacylglycerols and 50% of triacylglycerols. All these mixtures can be utilized as self-emulsifying vehicles for the formulation of bioactive substances and also as precursors for the production of structured bioactive lipids.

## Introduction

In recent years, partial glycerides, such as diacylglycerols (DAG) or monoacylglycerols (MAGs), are achieving increasing popularity from different perspectives. On the one hand, the most popular function of these lipids is related to their amphiphilic nature and surface-active properties, being well known as emulsifier ingredients in the food, pharmaceutical, and cosmetic industries. On the other hand, partial glycerides, such as self-emulsifying lipid delivery systems, are attractive lipids in the formulation of potential vehicles of drugs and bioactive compounds of poor solubilization, in order to favor their bioaccessibility at intestinal level or to protect against their degradation under gastrointestinal (GI) conditions, and ultimately to reach the most efficient bioactivity of compounds (Martin et al., 2014). Besides, the development of lipid structures containing bioactive fatty acids has additional relevance based on the increasing interest in incorporating omega-3 PUFA into the diet driven by the extensive literature regarding the promotion of health and disease prevention of these dietary PUFA. Numerous studies, including *in vitro* cell-based and animal-feeding studies, as well as observational studies and randomized controlled trials in humans, support these claims (Surette, 2013). In this sense, Echium oil, a natural source of stearidonic acid (C18:4 *n*-3) has been the subject of increasing attention as an effective precursor of long-chain PUFA (Whelan, 2009; Baik et al., 2014, 2015).

Oil represents one of the most important excipients in emulsifying formulations because it can solubilize marked amounts of the lipophilic drug, facilitate emulsification, and increase transport and absorption of lipophilic drug via GI tract. Molecular nature of the triglyceride plays an important role, and surprisingly, very few lipid-based formulations have reached the pharmaceutical market place (Gupta et al., 2013). This may be due to the insufficient information regarding the physical chemistry properties of lipids and concerns about their chemical and physical stability. However, edible oils which represent the logical lipid excipient for the development of drug delivery system are not frequently selected due to their poor ability to dissolve large amounts of lipophilic drugs (Hauss et al., 1998). On the contrary, modified or hydrolyzed vegetable oils form good emulsification systems and have been widely used for this matter. In addition to their formulation advantages, their degradation products resemble the natural end products of intestinal digestion. Thus, the impact of any lipid-based formulation on the lipid digestion processes must be considered. In this sense, it should be also mentioned that acylglycerols mixtures might be favorable in terms of absorption and utilization efficiency for some bioactive fatty acids (Banno et al., 2002). Hence, these modified or hydrolyzed oils may be beneficial for both the poorly soluble bioactive ingredient and the bioactive fatty acid that in some situations could have synergetic effect.

One of the tools available for the production of mono- and diacylglycerol mixtures is solvent-free glycerolysis, which is a simple process and easily scalable. In this procedure, there is no production of free fatty acids (FFAs) that otherwise, should be removed at the end of the process. No organic solvents are utilized which avoids time-consuming, costly solvent recovery and increases the acceptability of the final product for food applications and for the development of emulsifying systems (Kristensen et al., 2005).

Several glycerolysis systems utilizing or not organic solvents, with immobilized or non-immobilized enzymes, and in microemulsion or other media have been reported. A common strategy called solid-phase reaction consists of carrying out the glycerolysis reaction below the critical temperature (*T*_c_), in which MAG is instantaneously crystallized to shift the reaction equilibrium toward the production of MAG. However, slow reaction rates (up to a week) and difficult reuse of enzymes in these systems make them not very practical from an industrial point of view (Yang et al., 2005). Excluding solid-phase glycerolysis, it has been reported that temperature is not crucial for reaction equilibrium in enzymatic glycerolysis reactions, which is especially relevant for those thermolabile polyunsaturated oils (Yang et al., 2005).

Alternatively, incorporation of surfactants may improve homogeneity, increase the interfacial area, provide higher reaction rates, and enhance the efficiency of the biocatalyst for the system oil/glycerol/enzyme. This is especially relevant for lipases which are characterized as enzymes that act at the interface. The characteristic of a surfactant is the formation of micellar systems that (1) increases enzymatic stability at the process conditions, (2) reduces mass transfer limitations, and (3) improves reaction conversion due to the increased solubility among substrates (Valério et al., 2010).

It is well known that glycerol has a negative impact on lipase activity and stability by being adsorbed onto the support of the immobilized lipases reducing the diffusion of the hydrophobic substrate to the active site of the lipase (Fureby et al., 1996; Dossat et al., 1999; Ferreira-Dias et al., 2001). This undesirable effect of glycerol reduces the operational stability of the enzyme and has a negative impact on the economic viability of the process. Speed and type of agitation, enzyme carrier chemical nature, and even the sequence of addition of the different substrates and enzyme to the reaction mixture could have a role in catalyst clogging (Xu et al., 2011). Moreover, it has been also reported that molar ratios glycerol to oil higher than 1 are not relevant on the equilibrium level reached, especially when hydrophobic carriers for the lipase are used and hence, excess of glycerol is not necessary (Kristensen et al., 2005). In this sense, reducing as much as possible the glycerol content in the reaction mixture could alleviate the enzyme–glycerol coating.

**Scheme 1:**
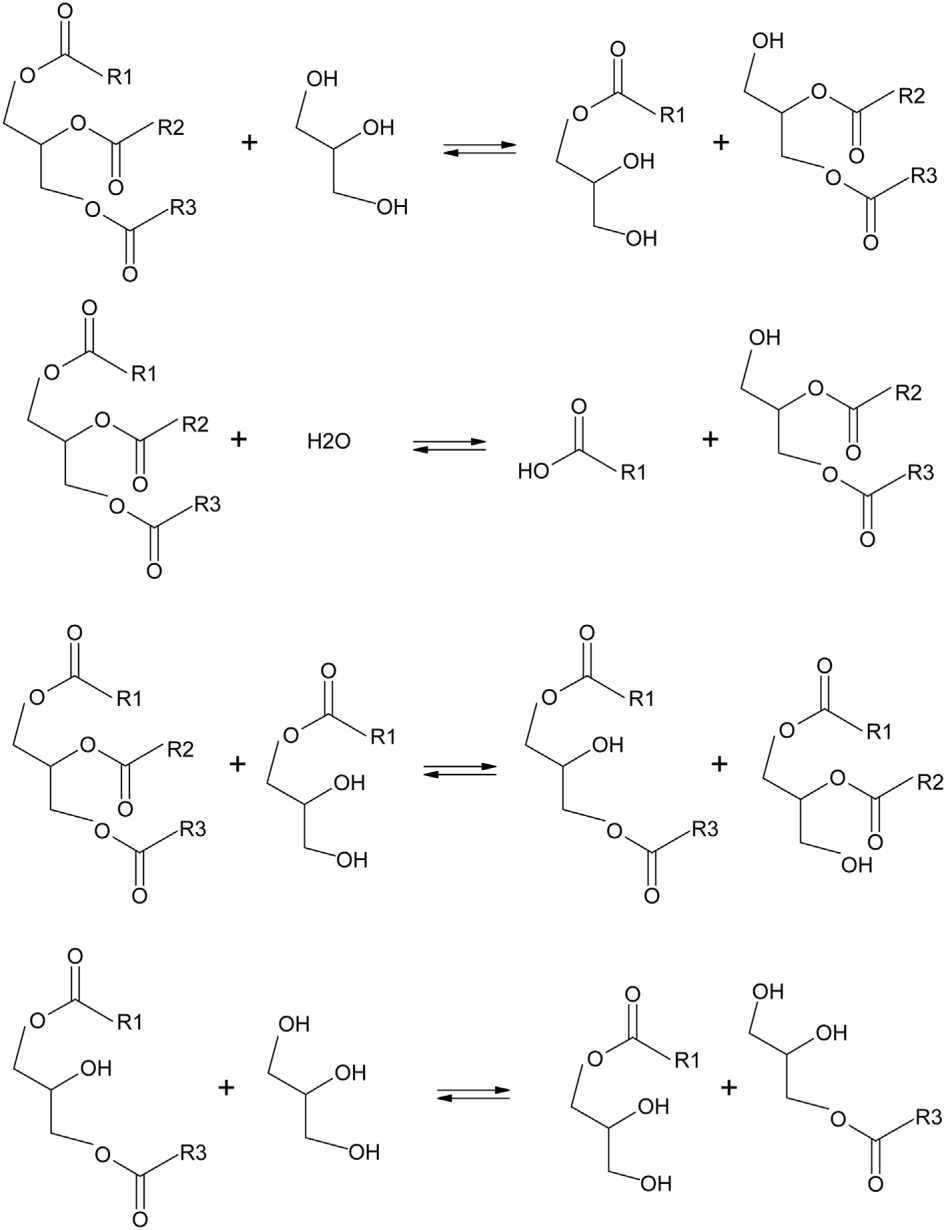
**Different enzymatic reactions involved in a process of glycerolysis**.

The ratio of 1,3- vs. 1,2-diacylglycerols is nutritionally important, and it has also implications in the product composition of glycerolysis reactions. It has been reported that non-specific lipases can remove fatty acid residues from sn-2 position of the triacylglycerol molecules forming 1,3-diacylglycerols. We also believe that other undesirable secondary reactions besides glycerolysis can take place to produce 1,3-diacylglycerols (Scheme 1). Transesterification reactions between triacylglycerols and sn-1 MAGs to produce 1,2- and 1,3-diacylglycerols can take place as soon as MAGs accumulate in the reaction mixture above the solubility limit of glycerol in the oily phase where the glycerolysis takes place (Kristensen et al., 2005).

The addition of organic solvents to the reaction mixture is one strategy to improve the system homogeneity and stability as well as to reduce viscosity and mass transfer limitations. However, even in glycerolysis reactions, organic solvent-assisted phase split behavior has been described (Damstrup et al., 2006). This phase split behavior does not indicate improved reactant miscibility in the solvent system compared to the solvent-free system. The enhanced reaction efficiency observed in the solvent system could be related to polarity changes of the system and reduced viscosity and mass transfer limitations of the reactant mixture. Moreover, the presence of an organic medium could produce a better emulsification of glycerol and oil with increased surface area accessible for the enzyme. It is also possible that this polarity changes prevent the adherence of glycerol to the enzyme, improving the contact between oil and enzyme. Finally, it should be noted that this phase split has been observed at short reaction times. The transformation of TAG and glycerol into MAG and DAG with emulsifying properties converts the final product into a homogeneous mixture (Damstrup et al., 2005, 2006; Yang et al., 2005).

In summary, considering the vast information found in the literature on glycerolysis reactions (Kaewthong et al., 2005; Damstrup et al., 2007) of different edible fats and oils, this study is the first attempt into the development of a potential bioactive acylglycerol mixture from Echium oil with emulsifying properties with capabilities to be used in the formulation of other bioactive substances and also, that can serve as precursors for the production of structured lipid after their fractionation via molecular distillation.

## Materials and Methods

### Materials

Echium oil was acquired to Harke Nutrition (Mülheim and der Ruhr, Germany). The fatty acid profile of the oil was comprised of palmitic acid (7%), stearic acid (4%), oleic acid (15%), linoleic acid (15%), gamma-linolenic acid (12%), alpha-linolenic acid (33%), and stearidonic acid (14%). The balance of the oil (1%) consisted of other minor fatty acids. Monoolein (99%), diolein (99%), triolein (99%), oleic acid (C18:1) (99%), and limonene were purchased from Sigma-Aldrich (MO, USA). All solvents utilized were HPLC grade acquired to Lab Scan (Giliwice, Poland). Novozym^®^ 435 and Lipozyme^®^ RM IM were a gift from Novozymes A/S (Bagsvaerd, Denmark). Lipase PLG (from *Alcaligenes* sp. immobilized on granulated diatomaceous earth) and not immobilized Lipase SL [from *Pseudomonas (Burkholderia) cepacia*] were purchased from Meito Sangyo Co., Ltd. (Tokyo, Japan). Monoolein food grade DIMODAN^®^ MO 90/D was purchased from Danisco (DuPont NHIB Iberica S.L., Barcelona, Spain).

### Glycerolysis Reactions

Ten grams of reaction mixture containing approximately 9 g of Echium oil and 1 g of glycerol were incubated in an orbital shaker (IKA KS 4000, Staufen, Germany) at 40°C and 200 rpm for 48 h. Echium oil and glycerol were preincubated for 10 min prior enzyme [10% (w/w) of the total reaction mixture] was incorporated to start the reaction.

At different times, aliquots of 50 μL were taken from the reaction mixture, dissolved in 5 mL of methyl tert-butyl ether (MTBE), and filtered through a syringe filter of 0.45 μm of pore size to remove the biocatalyst since all biocatalysts tested had a particle size higher than 1 μm. Aliquots from these solutions were taken and evaporated under nitrogen to obtain a constant weight residue that was redissolved in MTBE up to a final concentration of 6 mg/mL. Approximately 0.2 μL of this final transparent solution was analyzed by gas chromatography.

Solvent-assisted glycerolysis was carried out utilizing the same amount of Echium oil and glycerol utilized in solvent-free reactions but by incorporating ca. 8 g of food grade limonene.

### Homogenization of the Reaction Mixtures

Approximately 20 g of reaction mixtures with identical ratio of Echium oil to glycerol to that described in glycerolysis reactions section, but containing 2% w/w of food grade monoolein, were prepared and homogenized by using an Emulsiflex C5 from Avestin Europe GmbH (Weinheimer, Mannheim, Germany). Five passes at approximately 500 bar were performed with each of the mixtures tested.

### Analyses of the Reaction Mixtures

Separations were performed on a Hewlett-Packard 5890 series II gas chromatograph with on-column injection using a 7-m HP-5MS capillary column, 0.25 mm I.D. (Agilent Technologies, Santa Clara, CA, USA). An injector and detector temperatures of 40 and 340°C, respectively, were utilized. The temperature program was as follows: starting at 40°C and then heating to 250°C at 42°C min^−1^ with 15 min hold, followed by heating from 250 to 325°C at 15°C min^−1^ with 20 min hold. Calibration curves for MAGs, diacylglycerols, triacylglycerols, and FFAs were carried out with monoolein (99%), diolein (99%), triolein (99%), and oleic acid (C18:1) (99%), from Sigma-Aldrich (MO, USA). The peaks were computed using GC chemstation software.

### Scale-up

Glycerolysis reactions were also carried out in a 1-L stainless steel reactor coupled to a paddle stirrer at 200 rpm (Kiloclave, Buchi Glass Uster, Switzerland) in the presence of Novozym 435 and PLG. Three consecutive trials reutilizing the same batch of each lipase were performed. The reaction mixture consisted of 500–600 g of Echium oil and ca. 10% of glycerol (based on the total weight of oil). After 10 min of preincubation at 40°C, 10% (w/w) of each lipase was incorporated to the reaction mixture. Both immobilized lipases were recovered at the end of the glycerolysis reaction by passing the product mixture and the biocatalyst through a filtering device with a 100-μm mesh to retain the enzyme inside the filter. This device was connected to the discharge valve of the reactor and the mixture was forced to pass through the filter with the help of nitrogen pressure (1–2 bar). The fluctuations on the amount of Echium oil utilized were based on the weight of the solid material recovered after filtering the product mixture. This solid material contained the recovered lipase impregnated of the product mixture, after each trial. As an example, more than 200 g of Novozym 435 impregnated in the product mixture from the first trial were recovered, and for this reason only, 500 g of Echium oil were utilized in the second trial with the mentioned impregnated Novozym 435. After 24 h, the reactions were finalized and the mixture was forced to pass through a 100-μm stainless steel filter with the help of 1–2 bar of nitrogen gas to separate the enzyme from the oily mixture.

### Wiped-Film Short-Path Distillation

The molecular distiller pilot plant used in this project was acquired from POPE Scientific Inc. (Saukville, WI, USA). An external condenser and a cryogenic trap was installed immediately downstream of the still. The condensable, low-molecular-weight compounds are collected in the cryogenic trap upstream of vacuum system. Molecular distillation was carried out at 220°C and pressure of ca. 0.005 bar at a flow rate of ca. 250 mL/h. The methodology utilized with slight modifications has been utilized for the production of distilled MAGs (Fischer, 1998).

## Results and Discussion

It is well known that the solubility of glycerol in the triglyceride at the reaction temperature is the determining factor for the yield of MAGs. Sonntag in 1982 reported a high yield of MAG in the glycerolysis reactions in different systems ranging from fine dispersion to superemulsion. Glycerol particle size of 10–0.05 μm and smaller, yields of 61–95% for MAGs. In all the cases examined, the reaction systems are still heterogeneous, although at superemulsion range, homogeneity is almost reached. An interesting conclusion is that besides high temperature and solubility, a high degree of contact may also be a feasible path for a high yield of MAGs. Therefore, a high shear liquid–liquid device besides to the solubility effects as a consequence of an increase of process temperature is expected to have a positive effect on glycerolysis reactions. Consequently, faster reactions at lower temperatures are feasible. Moreover, some of the undesirable taste and color due to the decomposition of triglycerides at high temperatures may be eliminated (Noureddini, 1994). Based on this initial premise, solvent-free enzymatic glycerolysis of Echium oil was carried out in three different reaction mixtures: (1) high-pressure-homogenized mixtures containing 2% by weight of monoolein (Δ homogenization), (2) mixtures containing 2% of monoolein without homogenization treatment (Υ 2% monoolein), and (3) reaction mixtures without homogenization pretreatment and without monoolein addition (□ control).

### Glycerolysis Catalyzed by Novozym 435

**Figure 1:**
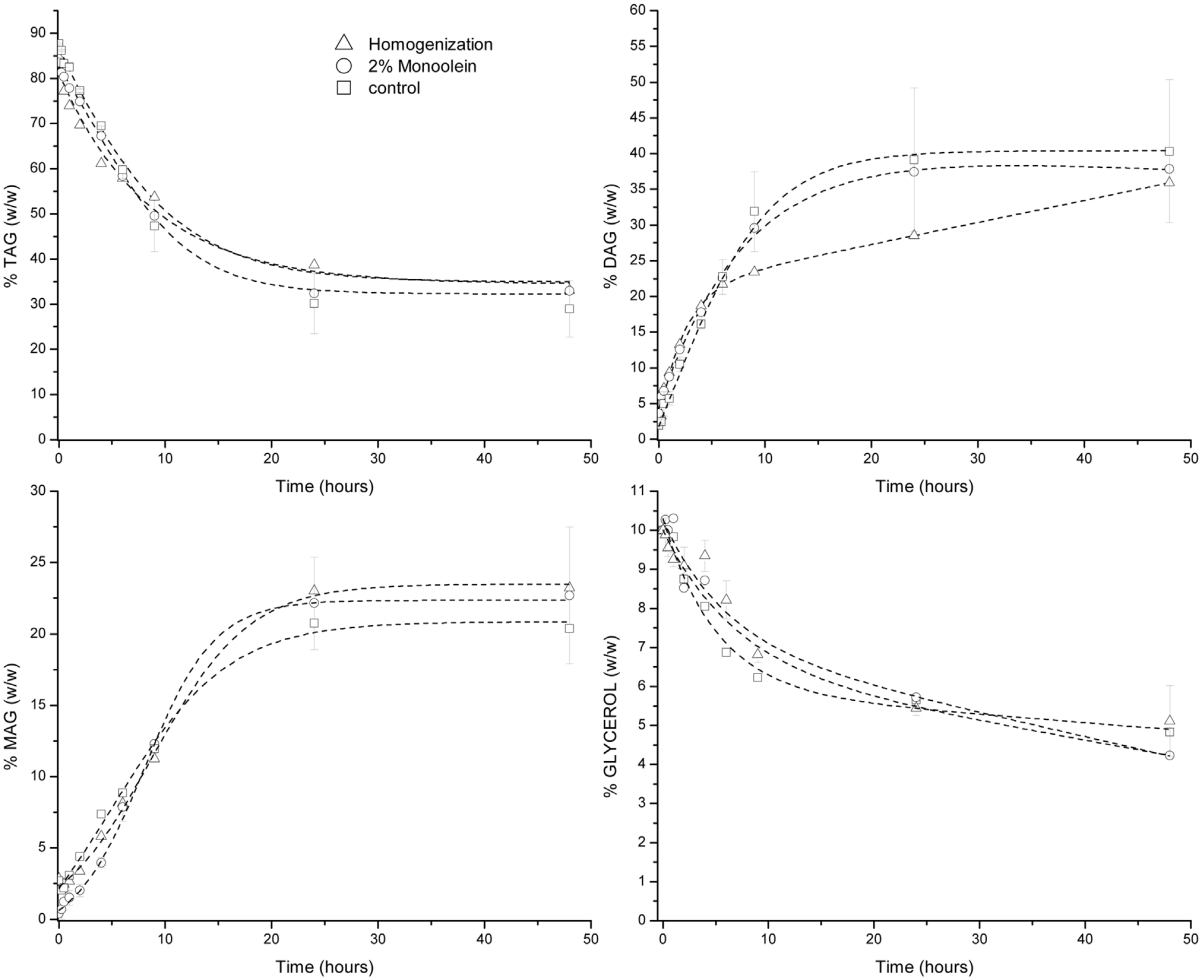
**Glycerolysis catalyzed by Novozym 435**.

The previously mentioned three different glycerolysis reactions were carried out in the presence of Novozym 435. The results are shown in Figure 1. The high pressure homogenization was performed in the presence of 2% (w/w) of food grade monoolein because homogenization attempts without monoolein did not produce a fine dispersion of glycerol in the oily phase. After addition of monoolein and five passes of homogenization, each one at 500 bar, glycerol phase was not visible in the reaction mixture. Then, the biocatalyst was added and the glycerolysis was incubated for 48 h. Similarly, reaction containing 2% of monoolein but without homogenization treatment was also carried out. These two enzymatic reactions were compared with the reaction in the absence of monoolein and without homogenization (glycerolysis control). The TAG conversion [ca. 65% (w/w)] was similar in the three cases under study. Glycerol consumption was determined based on the difference of total acylglycerols at different times and those at the beginning of the reaction. The calculated glycerol consumption was also very similar in the three cases under study. The main difference observed was the production of diacylglycerols that was slower in the homogenization treatment. The differences observed can be caused by different glycerol particle size, partial enzyme coating, slightly different degree of hydrolysis coupled to the glycerolysis reaction, etc. It has been also reported that not only glycerol but also its solutions with water may block the access of the hydrophobic substrates to the lipase active site, acting as inhibitors (Lai et al., 2005). For all these reasons, it results quite complicated to obtain complete reproducible results in a three-phase reaction system comprised of two immiscible viscous liquid phases and one solid phase (biocatalyst) that can even suffer from coating in different degrees by one of the liquid phases (glycerol phase) affecting the mass transfer and diffusion of the other liquid phase (oil) to the active site of the enzyme. Replicates of the control reaction were performed and the SDs of the different reaction times analyzed confirm the mentioned reaction rates fluctuation. Finally, it should be mentioned that the MAG content of the mixtures never exceeded 25% (w/w).

### Glycerolysis Catalyzed by RM IM

**Figure 2:**
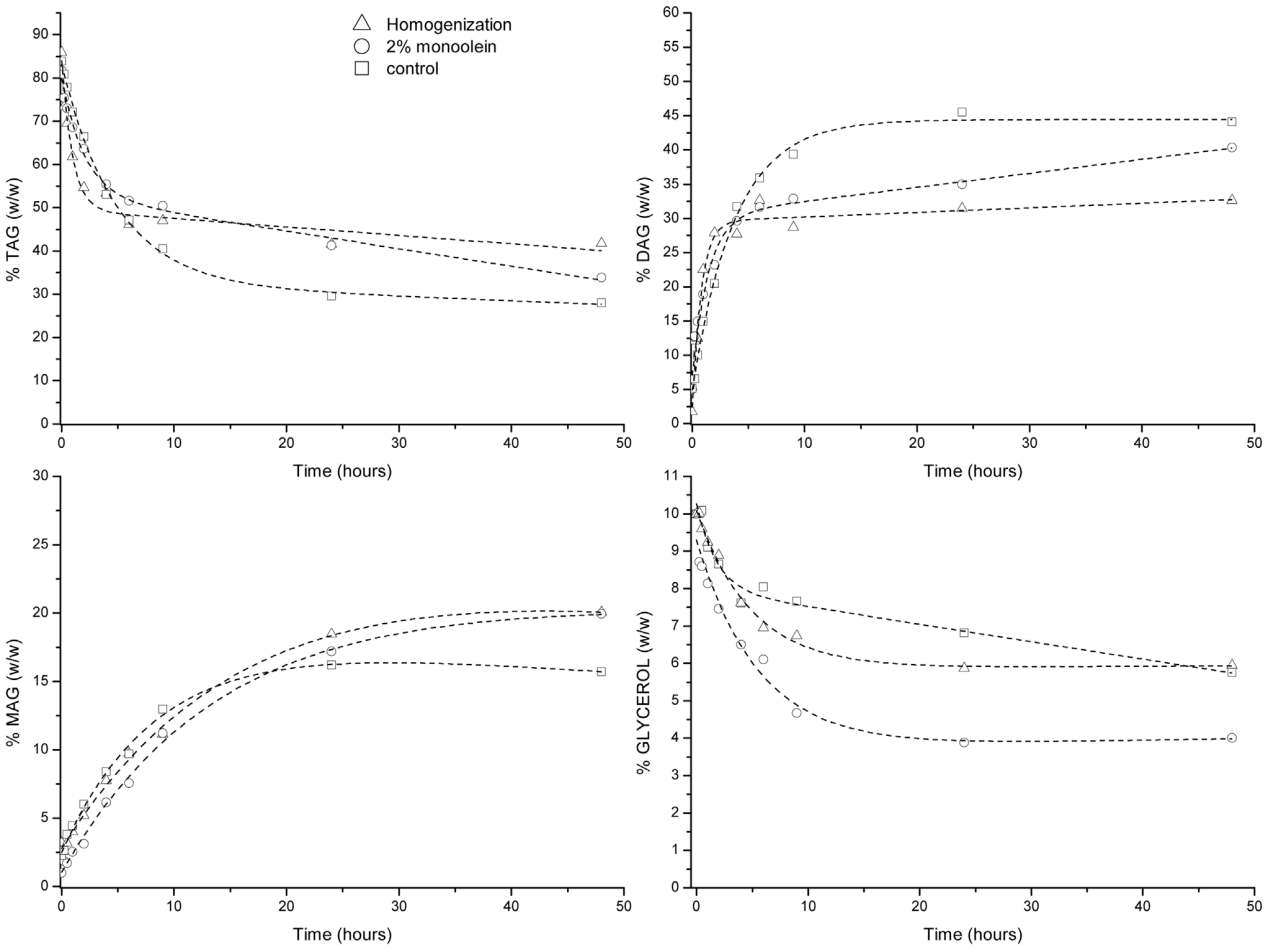
**Glycerolysis catalyzed by lipozyme RM IM**.

Similarly to the glycerolysis reaction in the presence of Novozym 435, three different glycerolysis reactions were also performed in the presence of lipase RM IM (Figure 2). TAG conversion oscillated between 60 and 70% for the three different types of glycerolysis reactions studied. The higher dispersion of results observed with this lipase could be attributed to glycerol coating of the immobilized enzyme, since apparition of enzyme aggregates after several hours of incubation regardless of the pretreatment utilized, were more noticeable with this enzyme than that with the rest of enzymes tested. In addition, slightly lower content of MAG (ca. 15–20% by weight) compared to Novozym 435 was produced after 48 h of glycerolysis reaction.

### Glycerolysis Catalyzed by PLG

**Figure 3:**
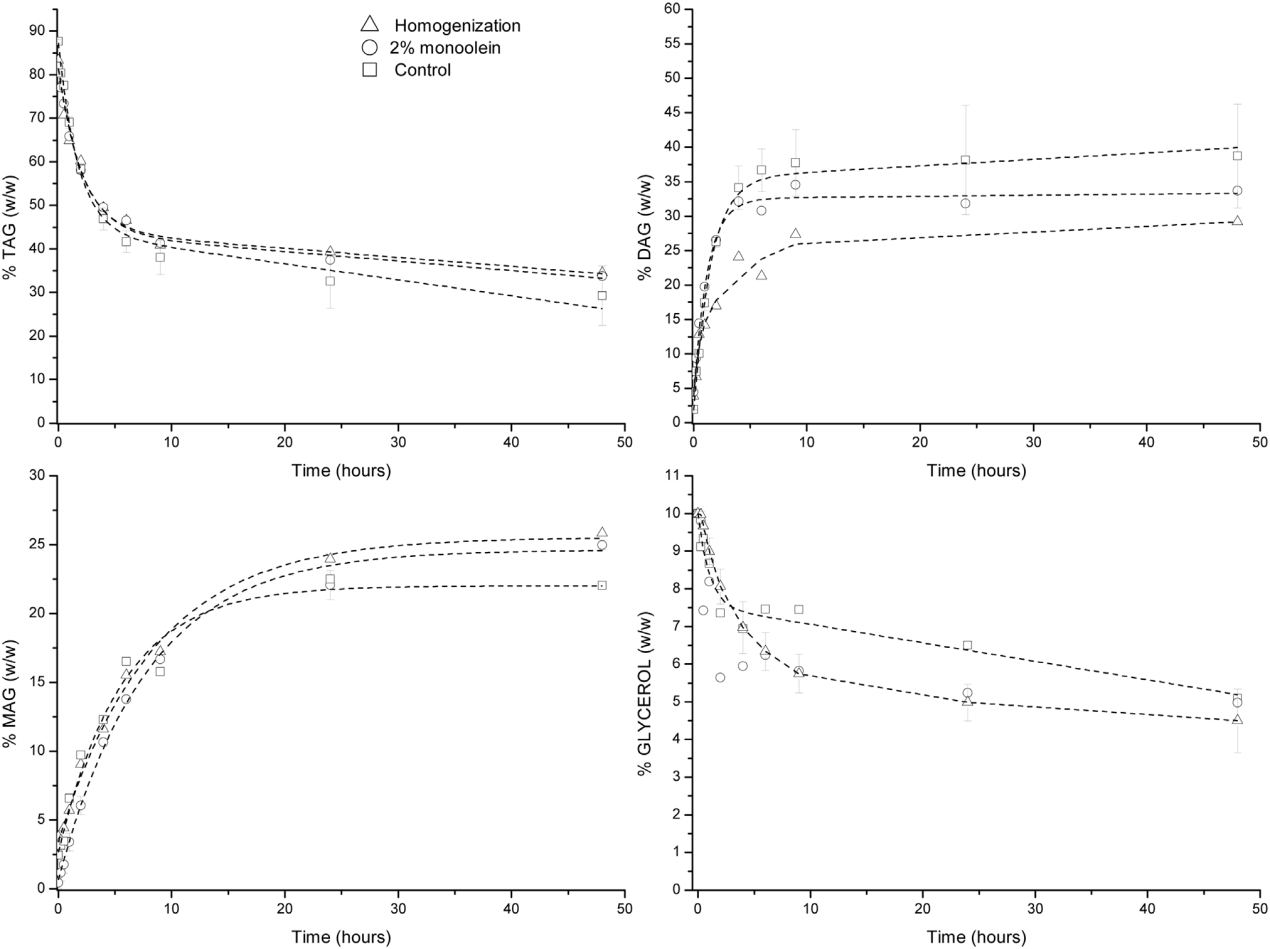
**Glycerolysis catalyzed by PLG (*Alcaligenes* sp.)**.

Similarly to the two previous lipases, in the presence of PLG lipase, regardless of the reaction mixture assayed, TAG and glycerol conversions were ca. 60–70 and 50%, respectively (Figure 3). In addition, diacylglycerol percentage fluctuated between 25 and 35% for the three different reaction mixtures investigated. The reasons for these differences are similar to those described for the glycerolysis in the presence of Novozym 435 and RM IM lipases. It should also be noted that MAG production was close to that attained in the presence of Novozym 435, but according to the initial reaction rates from the three mixtures assayed, they were higher in the presence of PLG than those observed with Novozym 435. Replicates of the control reaction were also carried out and the SDs of the different reaction times analyzed confirm the mentioned reaction rates fluctuation.

### Glycerolysis Catalyzed by Lipase SL

**Figure 4:**
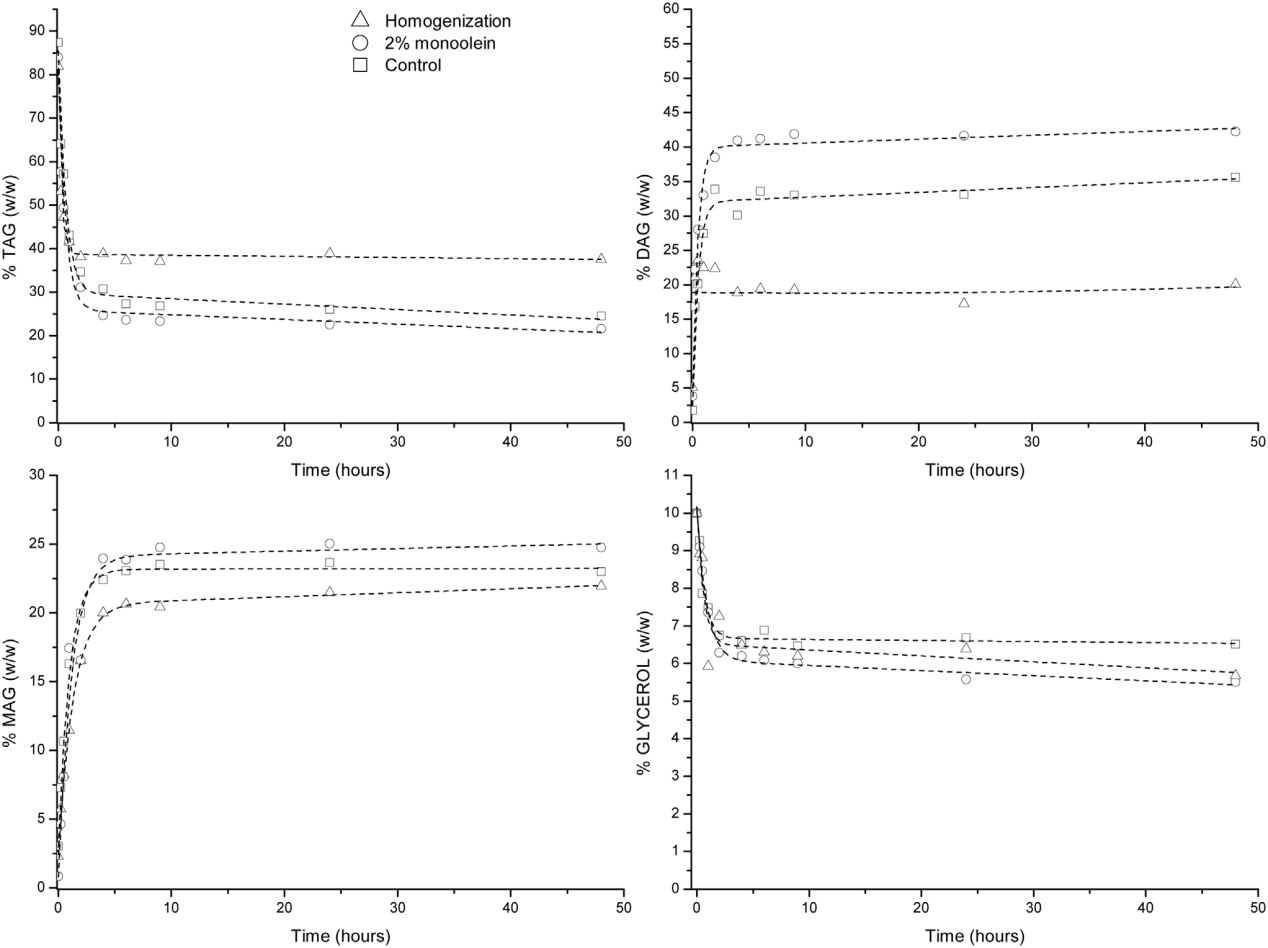
**Glycerolysis catalyzed by *Burkholderia cepacia***.

Regardless of the reaction mixture utilized, TAG conversion in the presence of *Burkholderia cepacia lipase*, fluctuated from 60 to 75% and surprisingly glycerol conversion calculated was, in average, lower than that with the other three lipases tested (Figure 4). This result indicates that the production of MAG and diacylglycerol takes place not only via glycerolysis but also via hydrolysis reactions. Diacylglycerol production ranged between 20 and 40% by weight in the presence of this lipase. These bigger fluctuations could be also attributed to different hydrolysis reactions coupled the glycerolysis reaction in the different pretreatments performed. In this case, FFA content of the product mixture was determined, and it oscillated between 9 and 13% and could be, in part, responsible of the differences observed.

It should be also noted that the highest reaction rates were observed with this lipase. This result could be attributed to the absence of carrier in this commercial lipase that reduce particle size, increase the surface area, and reduce diffusion and mass transfer limitations. However, considering the high FFA content in the product mixture, glycerolysis in the presence of Lipase SL was not further investigated because it requires complicated purification steps to remove the released FFA from the acylglycerol mixture.

It can be concluded that high pressure homogenization or the addition of 2% of monoolein to the reaction mixtures did not offer any significant advantage over the glycerolysis reaction. On the contrary, in some cases, after homogenization, lower conversions were even observed that could be attributed to different reasons: glycerol particle size distribution, partial enzyme coating, slightly different degree of hydrolysis coupled to the glycerolysis reaction, enzyme carrier polarity, etc. Solvent-free glycerolysis takes place in a very heterogeneous reaction media, and it is affected by numerous factors that influence enzyme kinetics and reproducibility.

As it has been already previously emphasized, one of the most important factors in glycerolysis is the solubility and the surface contact between oil and glycerol. In this sense, in chemical glycerolysis, the catalyst form soaps, which promotes the reaction acting as emulsifiers. Similarly, the high temperature reduces mass transfer of the triglyceride to the glycerol phase, increases the mutual solubility of fat and glycerol phases, and conducts to a faster rate of reaction. However, decomposition of some fatty acids at high temperatures prevents utilization of temperatures above 250°C. Unfortunately, this strategy is inadequate for oils rich in polyunsaturated fatty acids because of the thermal instability caused by double bonds in the carbon chain (Noureddini, 1994).

Based on the triacylglycerol conversion, production of mono- and diacylglycerols, and hydrolysis level of the glycerolysis reactions, we chose Novozym 435 and PLG lipases without any pretreatment for further experiments.

One important issue in this type of reaction is the stirrer utilized in the process. In our opinion, mechanical stirrers should produce a much more homogeneous reaction mixture and hence, partially overcome some of the limitations pointed out in the present study. However, care should be taken with some of these mechanical stirrers, particularly magnetic stirrers, at lab scale. These devices frequently produce mechanical abrasion of the biocatalyst reducing its particle size, which change the catalyst properties, reduce reusability and half-life of the enzyme. Moreover, the results attained with magnetic stirrers are not always readily scalable. For these reasons, we have chosen an orbital shaker incubator despite the limitations and fluctuations described above.

### Influence of Temperature in Solvent-Free Enzymatic Glycerolysis

**Figure 5:**
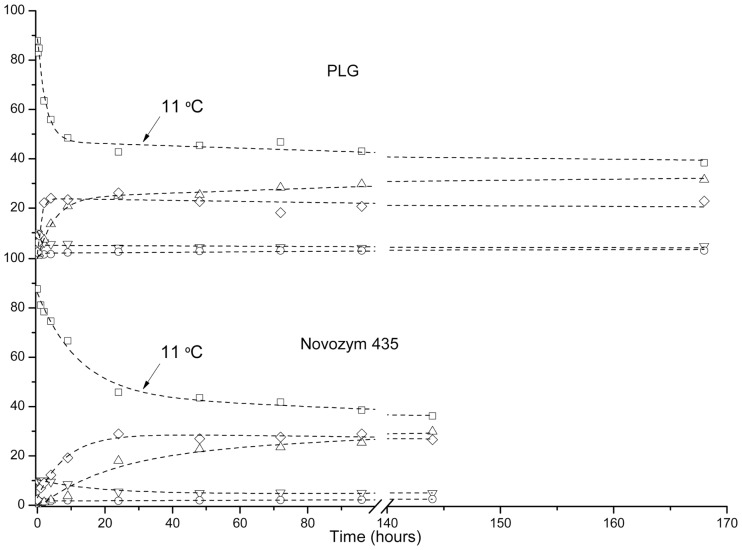
**Influence of temperature in glycerolysis of Echium oil**. ▽ Glycerol, ◯ free fatty acid, Δ MAG, ◊ DAG, □ TAG.

In order to precipitate MAGs in the reaction mixture and shift the reaction equilibrium toward the formation of more products, glycerolysis reactions were carried out in two stages in the presence of Novozym 435 and PLG lipases. The first stage of 24 h at 40°C was followed by a second stage at 11°C. The results shown in Figure 5 indicate that glycerolysis at 40°C in the presence of PLG is faster than in the presence of Novozym 435. The reduction of the temperature from 40 to 11°C slightly increases the MAG content from ca. 25 to 30% after 168 h of glycerolysis reaction in the presence of PLG and from ca. 18 to 30% in 144 h of glycerolysis reaction in the presence of Novozym 435. Diacylglycerol content was kept almost constant in the course of the reaction after 4 and 24 h in the presence of PLG lipase and Novozym 435, respectively. However, the reaction mixture transformed into a semisolid material that significantly reduced the reaction rate and also made difficult enzyme recovery at the end of the bioprocess. Moreover, after 168 h and 144 h triacylglycerol conversion was ca. 60% for both biocatalysts. For these reasons, this strategy was discarded.

### Solvent-Assisted Glycerolysis

**Figure 6:**
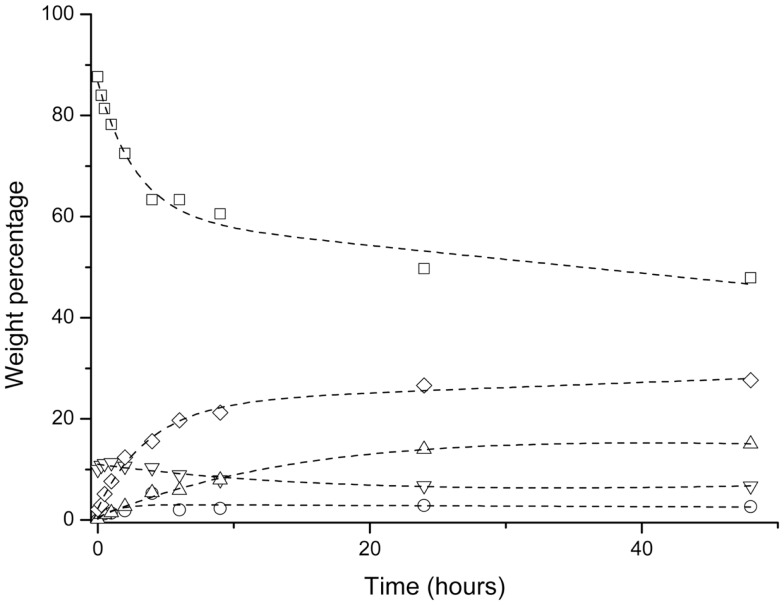
**Enzymatic glycerolysis in the presence of limonene**. ▽ Glycerol, ◯ free fatty acid, Δ MAG, ◊ DAG, □ TAG.

The enzymatic glycerolysis carried out in limonene is shown in Figure 6. Limonene is one of the most common terpenes in nature. It is a major constituent in several citrus oils (orange, lemon, mandarin, lime, and grapefruit). d-Limonene is listed in the Code of Federal Regulations as Generally Recognized As Safe (Grasso et al., 2007) for a flavoring agent and can be found in common food items such as fruit juices, soft drinks, baked goods, ice cream, and pudding. d-Limonene is considered to have fairly low toxicity. For these reasons, it was chosen as a solvent to perform the enzymatic glycerolysis. The *Log P* of this solvent is quite high (3.5), which indicates that is not very suitable for glycerol solubilization purposes. However, two-phase systems in the presence of a much more polar solvent, such as tert-butyl alcohol (*Log P* 0.75), have been also reported (Damstrup et al., 2005). Our initial hypothesis was that the presence of a solvent in the mixture could reduce the viscosity and mass transfer limitations, and thereby, improve the contact between enzyme and reactants. For this reason, we decided to carry out the glycerolysis in the presence of limonene utilizing Novozym 435 as biocatalyst. However, the results depicted in Figure 6 indicate lower conversion and lower content in MAGs in the product mixture even after 48 h of glycerolysis reaction. Moreover, it could be also pointed out that limonene could produce a dilution effect on both, the reaction mixture and the enzyme loading, therefore reducing the reaction rate. For these reasons, this strategy was discarded.

### Scale-up of Glycerolysis Reaction

Three consecutive trials with Novozym 435 and with PLG reusing the same batch of enzyme were carried out. The results are shown in Table 1.

**Table 1:** **Composition of the pilot plant glycerolysis trials carried out in the presence of PLG and Novozym 435**.

Biocatalyst	Trial number	Time (h)	MAG% (w/w)	DAG% (w/w)	TAG% (w/w)
PLG	1	24	22.1	29.5	47.7
PLG	2	24	11.6	30.2	58.0
PLG	3	24	4.1	25.8	66.7
Novozym 435	1	24	27.2	29.4	41.9
Novozym 435	2	24	27.5	30.7	41.0
Novozym 435	3	24	27.2	31.1	38.5

*Trial number refers to the three consecutive trials that were carried out reusing the same batch of lipases PLG and Novozym 435*.

The first trial with Novozym 435 produced slightly higher level of MAGs compared with the results at lab scale, which indicates that mechanical stirring can have a positive effect in glycerolysis compared to orbital shaking at lab scale. On the contrary, worse MAG production than that attained at lab scale was observed in the first trial with PLG. As it was mentioned before, partial coating of the enzyme could be also responsible of these differences.

Surprisingly, the second trial reutilizing the same batch of PLG produced much worse triacylglycerol conversion and percentage of MAGs than those obtained in the first trial. Physical appearance of the enzyme after the second trial was very different from the first trial. Enzyme aggregates of several millimeters could be observed in the recovered lipase, which could be responsible of the worse results obtained. To overcome this problem, the immobilized enzyme was washed twice with ethanol 95% and dried by vacuum filtering to breakdown the formed aggregates and to recover the batch of enzyme in a state similar to that of the first trial. With this washed and dried lipase PLG, a third trial was carried out. Unfortunately, as soon as the enzyme got into contact with the reaction mixture, similar aggregates to those observed in the second trial were formed. The results of the third trial indicate even worse triacylglycerol conversion and MAG production than those reached in the second trial that could be attributed to a more severe coating and also to partial inactivation of the biocatalyst after the washing and drying treatment. On the contrary, similar triacylglycerol conversion and MAG production was attained in the presence of Novozym 435 in the three consecutive trials studied. For this reason, this biocatalyst was chosen as the most suitable for the solvent-free glycerolysis of Echium oil.

### Molecular Distillation

Approximately 1.5 kg of the product mixture attained in the pilot plant glycerolysis trials described before was utilized for fractionation via molecular distillation. The feed material and the composition of the two products obtained after fractionation are depicted in Table 2. Molecular distillation of acylglycerol mixtures is a well-known procedure broadly utilized for the production of distilled MAGs. Usually, it is carried out at 200°C and vacuum levels of ca. 0.01 mbar (Bethge, 2014). In our trial, a first degassing pass at 100°C and 0.01 mbar was carried out to remove all volatile compounds. After this first pass, the cold trap contained <100 g of a viscous material comprised mainly of unreacted glycerol. Then, a second pass at 220°C and ca. 0.005 mbar was carried out to completely remove MAGs and FFAs from the residue fraction. The distillation was carried out at a flow of 250 mL/h and 390 g of a distillate containing ca. 80% of MAGs was obtained. One thousand seventy-seven grams of a residue comprised mainly of di- and triacylglycerols were also obtained. These two products can be utilized for different purposes. As an example, highly purified MAGs can be utilized as emulsifier and also as a precursor of structured lipids. The residue fraction can be utilized in a subsequent glycerolysis reaction to produce new mono-, di-, and triacylglycerol mixtures and also combined with appropriate amounts of MAGs as a self-emulsifying vehicle for bioactive ingredients.

**Table 2:** **Feed material and composition of the two products obtained after fractionation via molecular distillation**.

	Feed material	Residue	Distillate
Weight (g)	1543	1077	390
FFA (%)	1.0	0.0	5.2
MAG (%)	24.3	2.1	79.7
DAG (%)	28.3	47.8	11.1
TAG (%)	41.0	49.1	6.0

## Conclusion

Solvent-free glycerolysis of Echium oil produced an acylglycerol mixture comprised of ca. 25% of MAGs, 30–40% of diacylglycerols and ca. 40% of triacylglycerols in the presence of PLG and Novozym 435 lipases, regardless the pretreatment performed on the mixture. Neither the high pressure homogenization, nor the addition of monoolein improved the results of the enzymatic glycerolysis. The reduction of the temperature transformed the mixture into a semisolid product reducing significantly the reaction rate. Slightly improvement of the MAG content was attained in the reactions carried out at 11°C. However, the long reaction times and the difficulties to recover the enzyme from the product mixture ruled out the utilization of this methodology. Lower conversion of the oil was obtained in the solvent assisted glycerolysis reaction. Scale-up of the solvent-free glycerolysis with PLG lipase revealed apparition of enzyme aggregates when the biocatalyst is reutilized even after ethanol washing of the enzyme to remove the product mixture from the biocatalyst. On the contrary, similar results of the reaction were obtained in the presence of Novozym 435 after three consecutive trials reutilizing the same batch of enzyme. Finally, molecular distillation produced two main fractions: a distillate is comprised of 80% of MAGs and a residue containing ca. 50% of both diacylglycerols and triacylglycerols. These products and fractions are intended to be used as bioactive acylglycerol mixture with emulsifying properties and also as precursors of other bioactive structured lipids.

## Conflict of Interest Statement

The authors declare that the research was conducted in the absence of any commercial or financial relationships that could be construed as a potential conflict of interest.
